# Notch2 signal is required for the maintenance of canine hemangiosarcoma cancer stem cell-like cells

**DOI:** 10.1186/s12917-018-1624-8

**Published:** 2018-10-03

**Authors:** Keisuke Aoshima, Yuki Fukui, Kevin Christian Montecillo Gulay, Ochbayar Erdemsurakh, Atsuya Morita, Atsushi Kobayashi, Takashi Kimura

**Affiliations:** Laboratory of Comparative Pathology, Department of Clinical Veterinary Sciences, Faculty of Veterinary Medicine, Hokkaido University, Kita 18 Nishi 9, Kita-ku, Sapporo, Hokkaido 060-0818 Japan

**Keywords:** Cancer stem cell-like cells, Hemangiosarcoma, Notch2, Oncology, Tumor Biology

## Abstract

**Background:**

Hemangiosarcoma (HSA) is a malignant tumor derived from endothelial cells which usually shows poor prognosis due to its high invasiveness, metastatic rate and severe hemorrhage from tumor ruptures. Since the pathogenesis of HSA is not yet complete, further understanding of its molecular basis is required.

**Results:**

Here, we identified Notch2 signal as a key factor in maintaining canine HSA cancer stem cell (CSC)-like cells. We first cultured HSA cell lines in adherent serum-free condition and confirmed their CSC-like characteristics. Notch signal was upregulated in the CSC-like cells and Notch signal inhibition by a γ-secretase inhibitor significantly repressed their growth. Notch2, a Notch receptor, was highly expressed in the CSC-like cells. Constitutive activation of Notch2 increased clonogenicity and number of cells which were able to survive in serum-free condition. In contrast, inhibition of Notch2 activity showed opposite effects. These results suggest that Notch2 is an important factor for maintaining HSA CSC-like cells. Neoplastic cells in clinical cases also express Notch2 higher than endothelial cells in the normal blood vessels in the same slides.

**Conclusion:**

This study provides foundation for further stem cell research in HSA and can provide a way to develop effective treatments to CSCs of endothelial tumors.

**Electronic supplementary material:**

The online version of this article (10.1186/s12917-018-1624-8) contains supplementary material, which is available to authorized users.

## Background

Hemangiosarcoma (HSA) is a malignant tumor derived from endothelial cells which commonly occurs in dogs (*Canis lupus familiaris*) [1]. Other animals and humans can have similar tumors but the occurrence is very rare [1, 2]. Most preferred sites in dogs are the liver, spleen, and right atrium of the heart [3–5]. Patients show poor prognosis due to its aggressive invasion to adjacent tissues, high metastatic rate, and blood loss from tumor ruptures [6, 7]. Surgical excision of tumor masses or affected organs and chemotherapy are the preferred treatment methods, however, survival time after treatment may not significantly increase [8, 9]. Furthermore, patients may suffer from severe side effects induced by chemotherapeutic drugs such as myelosuppression and cardiotoxicity, which can limit the survival time extension in treated humans and animals [10, 11]. Therefore, novel effective treatments which can selectively target neoplastic cells have been warranted for decades [9].

Tumors, in general, are composed of many types of cells at various differentiation states and form cellular hierarchy in which cancer stem cells (CSCs) are located at the top [12, 13]. CSCs are a source of neoplastic cells which form tumor masses and are also involved in tumor recurrence after surgical excision, chemotherapy, or radiotherapy [12, 13]. Therefore, CSCs can be a good therapeutic target to completely eliminate tumors [13–15]. Using adult stem cell markers for normal tissues, CSC-like cells have been identified from many types of cancers including leukemia, breast cancer, and melanoma [16–18]. CSCs of HSA, however, have not yet been identified and adult stem cell markers for endothelial cells are also still unknown.

Notch, a type I transmembrane protein, has four identified types (Notch1 to 4). Notch receives signals from neighboring cells expressing the ligands and transduces these signals by translocation of the intracellular domain into the nucleus [19, 20]. Notch signal is demonstrated to be highly involved in several biological events such as stem cell maintenance, cellular differentiation, angiogenesis, and tumorigenesis [21–24]. CSCs of several types of tumors use Notch signaling to communicate with tumor microenvironment in order to maintain their stemness [25–29]. Notch signal disruption has been reported to be associated with irregular vascular proliferation and vascular tumors in humans and mice [30–32]. Canine HSA is hypothesized to be regulated by the signal transduction, though the role of Notch in HSA has not yet been studied.

The aim of this study was to isolate CSC-like cells from canine HSA cell lines and investigate the role of Notch signaling in HSA CSC-like cells.

## Methods

### Cell culture

We used seven hemangiosarcoma cell lines (JuA1, JuB2, JuB4, Ud2, Ud6, Re12, and Re21) kindly given by Dr. Hiroki Sakai, Gifu University [33]. Human embryonic kidney 293 T cell line and HeLa cell line derived from human cervical cancer were purchased from RIKEN BioResource Research Center. These cells were cultured with Dulbecco’s Modified Eagle’s Medium (D-MEM; Wako, Osaka, Japan) supplemented with 10% fetal bovine serum (FBS; Biowest, UT, USA) and Penicillin/Streptomycin (Thermo Fisher Scientific, MA, USA). For serum free culture, cells were seeded in culture plates coated with 0.1% gelatin (Wako) and cultured with Dulbecco’s Modified Eagle’s Medium/Nutrient Mixture F-12 Ham (Wako) supplemented with 10 ng/ml basic fibroblast growth factor (bFGF; Wako), 20 ng/ml epidermal growth factor (EGF; Thermo Fisher Scientific), NS Supplement (Wako) and Penicillin/Streptomycin [34–37]. Cells in both conditions were maintained at 37 °C with 5% CO_2_ prior to use in experiments. Cells were maintained under serum-free condition at least 2 weeks prior to the experiments. Cells were stained with Trypan Blue (Thermo Fisher Scientific) to stain dead cells and only the unstained, viable cells were used for determining cell number.

### Notch signal inhibition

A γ-secretase inhibitor, N-[N-(3,5-Difluorophenacetyl)-L-alanyl]-S-phenylglycine t-butyl estel (DAPT; Wako), was added to culture medium and dimethyl sulfoxide (DMSO) was used as the control. To find out the appropriate DAPT concentration, expression levels of Notch signal target genes (*HES1* and *HEY1*) were analyzed in HSA cell lines treated with DAPT or DMSO for 48 h. To make the growth curves, 5 × 10^3^ cells were seeded in 12-well plates in triplicate and were cultured in the medium containing 20 μM DAPT or DMSO. The cell numbers were counted at each passage point followed by reseeding of 5 × 10^3^ cells into 12-well plates. This procedure was repeated three or four times and the relative cell number was counted as the cell number at each passage point normalized to the original seeding cell count.

### Reverse transcription quantitative polymerase chain reaction (RT-qPCR)

Total RNA was extracted with TriPure Isolation Reagent (Roche, Basel, Switzerland) according to the manufacturer’s instructions. Reverse transcription was performed using Primescript II 1st strand cDNA Synthesis Kit (Takara Bio, Kusatsu, Japan) according to the manufacturer’s instructions after treatment with DNaseI (Thermo Fisher Scientific) for 15 mins at room temperature (RT) followed by EDTA treatment for 10 mins at 65 °C. Sample preparation for qPCR was performed using KAPA SYBR FAST qPCR Kit Master Mix (2×) ABI Prism (KAPA Biosystems, MA, USA). Reaction solution contains 1× KAPA SYBR FAST qPCR Master Mix, 200 nM forward and reverse primers, 1 μl cDNA and UltraPure DNase/RNase-free distilled water (UPDW, Thermo Fisher Scientific). The samples were applied in triplicate and analyzed by StepOne Real-time PCR system (Thermo Fisher Scientific). UPDW and no RT samples were used as negative controls. We confirmed that no signal was detected in the negative controls for all samples. Samples were denatured at 95 °C for 3 min followed by 40 cycles of 95 °C for 3 s and 60 °C for 20 s. Results were normalized based on geometric mean of reference genes (*GAPDH*, *ACTB*, *HMBS*). Reference genes were selected from nine potential internal controls (*GAPDH*, *ACTB*, *B2N*, *HMBS*, *HPRT1*, *RPL13A*, *RPL32*, *TBP*, *YWHAZ*) by geNorm software [38, 39]. Primer sequences for qPCR are listed in Table 1. Ensembl and Primer3 softwares were used to design 80 to 150 bp primers which can target all splice variants and cross exon-exon junctions. The BLAST database and software were used to confirm that each primer sequence is not detected in other genes. The *HES1* primer and potential internal control primer set sequences were obtained from a journal article published elsewhere [39, 40]. Primer efficiency was calculated based on the slope obtained from each standard curve and was confirmed to be more than 90% for all primer sequences (Additional files 1 and 2: Figures S1 and S2). Primer set specificity was evaluated by checking that each primer set have identical and singular peak in the melting curve.

**Table 1:** Primer list for RT-qPCR

Primer	Sequence		Gene ID and references	Efficiency (%)
	Forward primer	Reverse primer		
*ERG*	CAAACATGACCACGAACGAG	AGGCCGTATTCTTTCACTGC	ENSCAFG 00000009912	98.4
*PROCR*	GCAGGAACACAATGCTTCAA	AAGATGCCTACAGCCACACC	ENSCAFG 00000007945	95.5
*SOX18*	TGAACGCCTTCATGGTGTG	GGCGTCAGCTCCTTCCAC	ENSCAFG 00000029278	94.5
*FLI1*	TACTGAACAAAGGCCCCAAC	ACTGTCCGAGAGAAGCTCCA	ENSCAFG 00000032412	96.9
*NOTCH1*	TACCGGCCAGAACTGTGAGGAGAA	GGAGGGCAGCGGCAGTTGTAAGTA	ENSCAFG 00000019633	93.6
*NOTCH2*	TCGGGATAGCTATGAGCCCT	GGCATGTTGCTTTCCCCAAC	ENSCAFG 00000010476	93.6
*NOTCH3*	ACAACTGCCAGTGTCCTCCT	GTCCAGCCATTGACACACAC	ENSCAFG 00000016107	93.9
*NOTCH4*	AAGCCCTGTCCACACAATTC	CTGGCATAGGGAAGAAGCTG	ENSCAFG 00000000791	95.4
*HEY1*	GCGCGGATGAGAATGGAAAC	GTCGGCGCTTCTCAATGATG	ENSCAFG 00000008391	95.3
*HEY2*	CGGCGAGATCGGATAAATAA	CGCGTCGAAGTAGCCTTTAC	XM_541232.5 ENSCAFG 00000032212	96.4
*HES1*	CATCCAAGCCTATCATGGAGA	GTTCCGGAGGTGCTTCACT	Dailey DD et al.	95.5
*HES6*	CAGGCCAAGCTGGAGAAC	GCATGCACTGGATGTAGCC	ENSCAFG 00000012428	96.5
*NRARP*	TGAAGCTGCTGGTCAAGTTC	CTTGGCCTTGGTGATGAGAT	ENSCAFG 00000019445	94.5
*FCER2*	GAGGAGGTGGAGAAGCTGTG	CCTCGCCGAAGTAGTAGCAC	ENSCAFG 00000030055	97.5
*GAPDH*	ATTCCACGGCACAGTCAAG	TACTCAGCACCAGCATCACC	ENSCAFG 00000015077	99.5
*ACTB*	CCAGCAAGGATGAAGATCAAG	TCTGCTGGAAGGTGGACAG	ENSCAFG 00000016020 Peters IR. et al.	98.8
*HMBS*	TCACCATCGGAGCCATCT	GTTCCCACCACGCTCTTCT	ENSCAFG 00000012342 Peters IR. et al	95.8
*RPL13A*	GCCGGAAGGTTGTAGTCGT	GGAGGAAGGCCAGGTAATTC	ENSCAFG 00000029892 Peters IR. et al	98.2
*RPL32*	TGGTTACAGGAGCAACAAGAAA	GCACATCAGCAGCACTTCA	ENSCAFG 00000004871 Peters IR. et al	95.9
*HPRT1*	CACTGGGAAAACAATGCAGA	ACAAAGTCAGGTTTATAGCCAACA	ENSCAFG 00000018870 Peters IR. et al	95.7
*B2M*	ACGGAAAGGAGATGAAAGCA	CCTGCTCATTGGGAGTGAA	ENSCAFG 00000013633 Peters IR. et al	98.2
*YWHAZ*	CGAAGTTGCTGCTGGTGA	TTGCATTTCCTTTTTGCTGA	ENSCAFG 00000000580 Peters IR. et al	93.4
*TBP*	ATAAGAGAGCCCCGAACCAC	TTCACATCACAGCTCCCCAC	ENSCAFG 00000004119 Peters IR. et al	97.3

### Colony formation assay (CFA)

One thousand cells were seeded in 6-well culture plates and were cultured until the diameter of the biggest colony reached 2 mm. Cells were fixed with 4% paraformaldehyde for 20 mins at RT and then stained with Crystal Violet (Sigma-Aldrich, MO, USA) for 30 mins at RT. After washing with phosphate buffered saline (PBS) and drying at RT, colonies were visualized using an inverted microscope (Eclipse TS100; Nikon, Tokyo, Japan) and colonies which have more than 50 cells were counted [41].

### Chemoresistance assay

Three (normal culture) or five (serum-free culture) thousand cells seeded in 96-well culture plates and were treated with either DMSO, doxorubicin (Wako) or paclitaxel (Wako) at increasing concentrations the following day. Soon after the treatments, culture medium in each cell lines were collected and the absorbance at the time of treatment (Tz) was measured. Seventy-two hours after treatment, cell viability was analyzed using Cell Counting Kit-8 (Dojindo, Kumamoto, Japan) according to the manufacturer’s instructions. The absorbance of each well 72 h after treatments (Ti) was measured at 450 nm using NanoDrop 2000 (Thermo Fisher Scientific). DMSO treated cells were used as the control (C). Growth inhibition rates were measured as: [(Ti-Tz)/(C-Tz)] × 100 for concentrations in which Ti>/=Tz, [(Ti-Tz)/Tz] × 100 for concentrations in which Ti < Tz [42].

### Aldehyde dehydrogenase (ALDH) assay

To analyze ALDH activity, ALDEFLUOR kit (STEMCELL technologies, Vancouver, Canada) was used according to the manufacturer’s instructions. We stained 5 × 10^5^ cells with ALDEFLUOR reagent for 50 mins at 37 °C. N,N-diethylaminobenzaldehyde (DEAB) was used to inhibit ALDH activity and DEAB treated cells were used as basis to gate ALDH positive population. The cells were analyzed using FACSVerse (Becton Dickinson, NJ, USA) after excluding the dead cells which were positive for 7-aminoactinomycin D (Thermo Fisher Scientific). The data was analyzed using FACSuite software (Becton Dickinson).

### Protein extraction and western blotting

Cells were lysed using Radioimmunoprecipitation buffer [RiPA buffer; 50 mM Tris-HCl (pH 8.0), 150 mM NaCl, 0.1% TritonX-100, 0.1% sodium dodecyl sulfate (SDS), 0.5% sodium deoxycholate, EDTA-free proteinase inhibitor cocktail (Sigma-Aldrich)]. Protein concentration was measured using Pierce BCA Protein Assay Kit (Thermo Fisher Scientific) according to the manufacturer’s instruction. Samples were denatured by adding 1/4 volume of 4 × Sample buffer [200 mM Tris-HCl buffer (pH 6.8), 8% SDS, 40% Glycerol, 1% bromophenol blue, 20% 2-mercaptoethanol] to each sample followed by incubation at 98 °C for 5 mins. Ten micrograms proteins were separated in 8% SDS polyacrylamide gels by electrophoresis and were transferred to polyvinylidene difluoride membrane (PVDF membrane: Merck Millipore, MA, USA) using Mini Trans-Blot Cell (BIO-RAD, CA, USA). Membranes were then blocked in 5% skim milk in Tris-buffered saline containing 0.05% Tween 20 (TBST) for 1 h at RT. Membranes were either incubated with anti-human Notch2 intracellular domain antibody (R&D systems, MN, USA; 1:2000), anti-FLAG M2 monoclonal antibody (Sigma-Aldrich; 1:1000) or anti-Actin antibody clone C4 (Merck Millipore; 1:10,000) overnight at 4 °C. After washing with TBST, membranes were incubated with donkey anti-goat IgG-HRP (Santa cruz, TX, USA; 1:5000) or ECL Mouse IgG HRP-linked whole antibody (GE Healthcare, IL, USA; 1:10,000) for 1 h at RT. After washing with TBST, signals were visualized with Immobilon Western Chemiluminescent HRP substrate (Merck Millipore) and detected by ImageQuant LAS 4000 mini (GE Healthcare). Images were processed with ImageJ software [43–45].

### Plasmid construction

Canine *NOTCH2* gene (ENSCAFG00000010476) cloned from the cDNA of Canine Aortic Endothelial Cells (CnAOEC; Cell Applications, CA, USA) was subcloned into a self-inactivating (SIN) lentiviral vector construct, CSII-CMV-MCS-IRES2-Bsd. To make the dominant negative form and constitutive active form of Notch2, 1–5343 bp and 5161–7413 bp of *NOTCH2* were amplified from full length *NOTCH2* gene, respectively [46]. These two mutants were also subcloned into the SIN lentiviral vector construct. FLAG sequences were added at the C-terminus of full length and mutant Notch2 constructs by inverse PCR.

### Lentivirus infection

We seeded 8 × 10^5^ 293 T cells in a 6 cm dish and cultured in antibiotic-free medium. Cells were transfected, using Lipofectamine 3000 (Thermo Fisher Scientific) according to the manufacturer’s instructions, with three constructs; a packaging construct (pCAG-HIVgp), a VSV-G and Rev expressing construct (pCMV-VSV-G-RSV-Rec) and SIN lentiviral vector constructs. Forty-eight hours after transfection, culture media containing the produced viruses were collected in 15 mL tubes and centrifuged at 6000 g for 16 h at 4 °C. Pellets were resuspended in normal culture medium and used as virus reagent. Cells were cultured in the virus reagent with 8 μg/ml Polybrene (Sigma-Aldrich). Eight hours after infection, the medium in cell culture wells was replaced with a fresh medium without the viruses. Forty-eight hours later, culture medium was changed to normal medium supplemented with 10 μg/ml Blasticidin for selection and the cells were maintained for future experiments.

### Immunohistochemistry (IHC)

Twelve canine HSA cases collected from Hokkaido University Veterinary Teaching Hospital were used for IHC. These cases were derived from the spleen, liver, kidney and thoracic cavity (Table 2). Tissue samples were processed routinely as described previously [47]. The slides were immersed in 10 mM sodium citrate buffer (pH 6.0), boiled for 15 mins in a microwave for antigen retrieval and then cooled down to RT. After washing with PBS, sections were treated with 0.3% H_2_O_2_ in methanol for 15 mins at RT to inactivate endogenous peroxidases followed by blocking with 10% rabbit normal serum (Nichirei biosciences, Tokyo, Japan) for 1 h at RT. Sections were incubated with anti-human Notch2 intracellular domain antibody (R&D systems; 1:40) for overnight at 4 °C. PBS instead of the primary antibody was added to the negative controls. After washing with PBS, sections were treated with biotinylated anti-goat IgG (Nichirei biosciences) for 1 h at RT followed by incubation with peroxidase conjugated streptavidin (Nichirei biosciences) for 10 mins at RT. After washing with PBS, signal detection was carried out by submerging the sections in freshly prepared solution of 3,3′-diaminobenzidine tetrahydrochloride (Dojindo, Kumamoto, Japan) for 5 mins, and the sections were counterstained with hematoxylin for 1 min and then dehydrated and mounted with cover glasses. Signals were captured with BX63 microscope (Olympus, Tokyo, Japan) and processed with ImageJ software.

**Table 2:** Case information

Case No.	Breed	Age	Sex	Location
1	Labrador retriever	10y	Spayed female	Spleen, Liver
2	Border Collie	13y	Male	Spleen
3	Maltese	10y	Male	Spleen
4	Scottish terrier	10y	Spayed female	Thoracic cavity
5	Miniature dachshund	11y	Female	Spleen, Liver
6	Golden retriever	9y	Spayed female	Spleen
7	Miniature schnauzer	11y	Male	Spleen
8	Golden retriever	9y	Castrated Male	Liver
9	Bichon frise	8y	Spayed female	Kidney
10	Labrador retriever	10y	Male	Spleen
11	Great pyrenees	10y	Castrated Male	Spleen
12	Golden retriever	9y	Male	Spleen

### Statistical analyses

For the comparison of gene expression between two samples, Student’s *t* test was performed. Dunnett’s test was used in comparing the effects of Notch2 and Notch2 mutant expressions with empty vector-infected cells as the control.

## Results

### HSA cell lines in serum-free culture condition have CSC-like characteristics

**Fig. 1:**
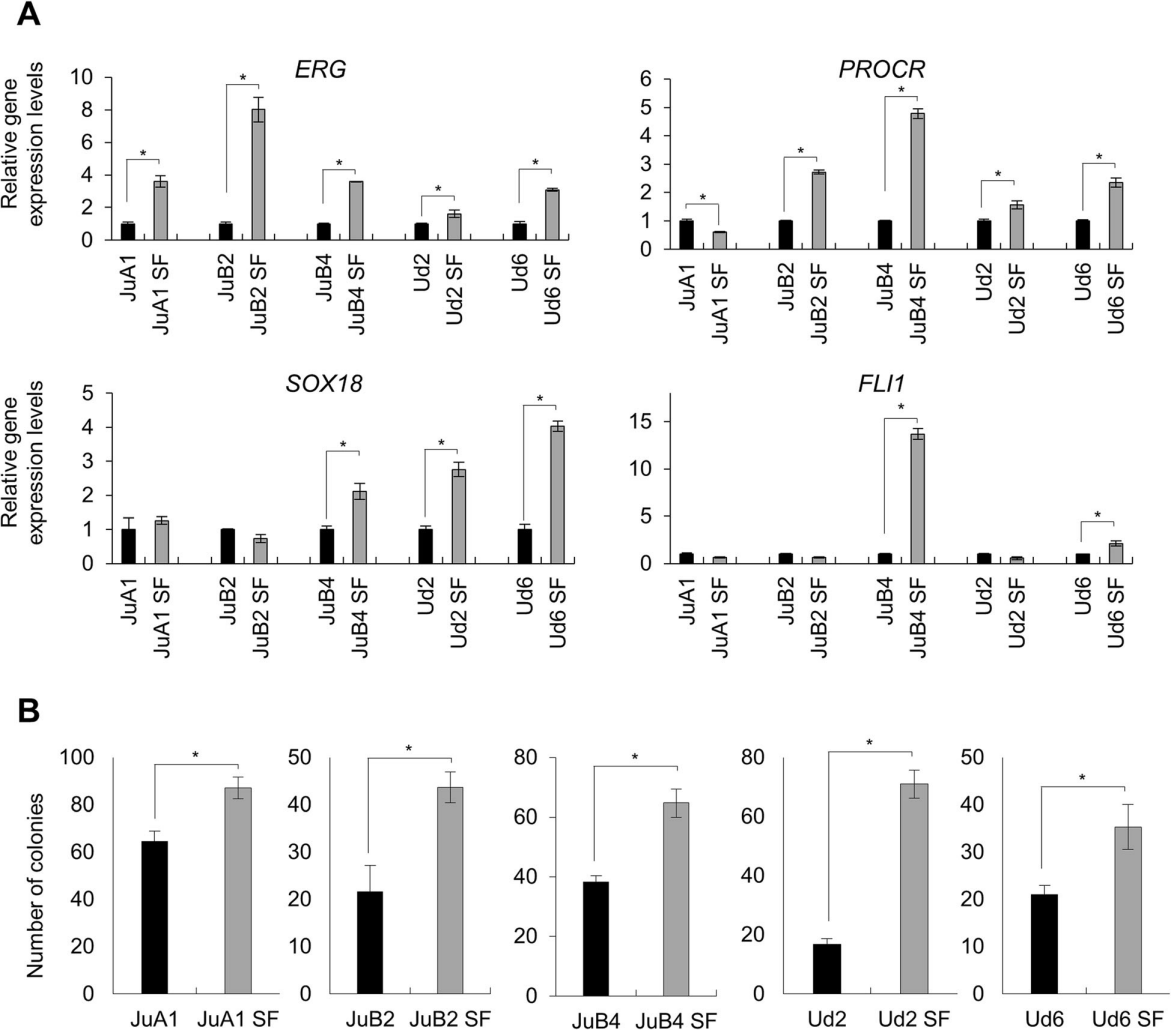
**a** Expression levels of undifferentiated endothelial cell-related genes in HSA cell lines cultured in normal or in SF conditions. Gene expression levels of each cell line in normal condition were set to 1. **b** The numbers of colonies of each cell line and condition. *p < 0.01. Student’s *t* test. All samples were analyzed in triplicates and the scores are presented as means ± SD

To isolate CSC-like cells from HSA cell lines, we cultured HSA cell lines in adherent serum-free (SF) culture condition in gelatin-coated cell culture plates. Approximately 70–90% Ju and Ud cells died within 2 days after culturing and surviving cells proliferated slowly. On the other hand, Re cells did not survive in this condition. Next, we checked expression levels of undifferentiated endothelial cell-related genes: *ERG*, *PROCR*, *SOX18* and *FLI1* (Fig. 1a) [48–50]. Prior to qPCR analysis, reference gene sets were selected from nine potential internal controls (*GAPDH*, *ACTB*, *B2M*, *HMBS*, *HPRT1*, *RPL13A*, *RPL32*, *TBP*, *YWHAZ*) using geNorm software (Additional file 3: Figure S3) [38, 39]. Based on the analysis, three reference genes (*GAPDH*, *ACTB*, *HMBS*) were selected and the geometrical mean of the expression levels of these genes was used as a control for normalization. *ERG* and *PROCR* were upregulated in all cell lines except for *PROCR* in JuA1. *SOX18* was highly expressed in JuB4, Ud2 and Ud6. *FLI1* was upregulated in JuB4 and Ud6. No significant repression of these genes was detected in SF condition except for *PROCR* in JuA1. We also analyzed the clonogenicity of HSA cells in serum-free condition using CFA. All cell lines cultured in SF condition had significantly increased number of colonies compared to cell lines cultured in normal condition (Fig. 1b).

**Fig. 2:**
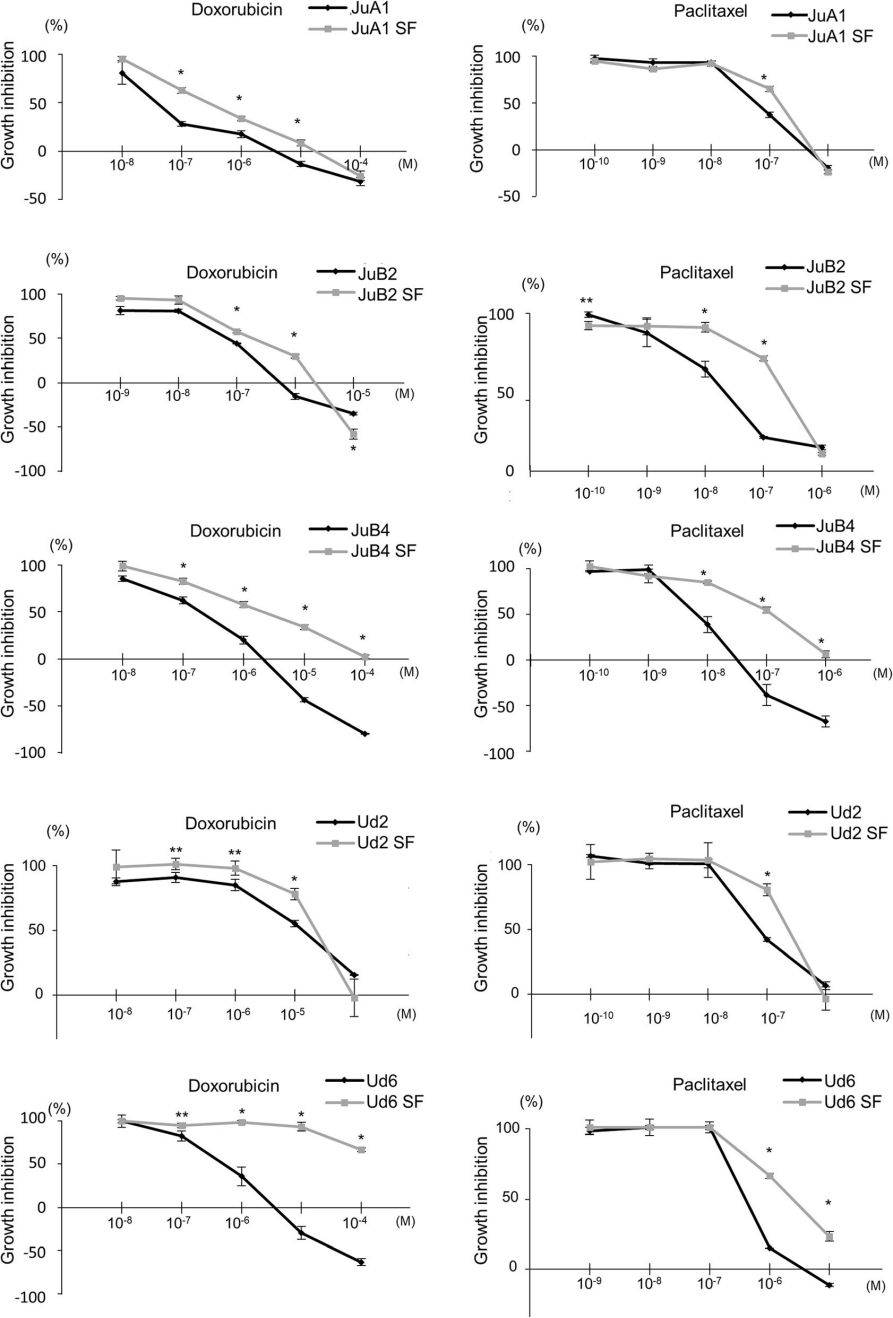
Survival rate of HSA cell lines treated with doxorubicin or paclitaxel. *p < 0.01. **p < 0.05. Student’s *t* test. All samples were analyzed in triplicates. Survival curves are plotted as average percentages ± SD

**Fig. 3:**
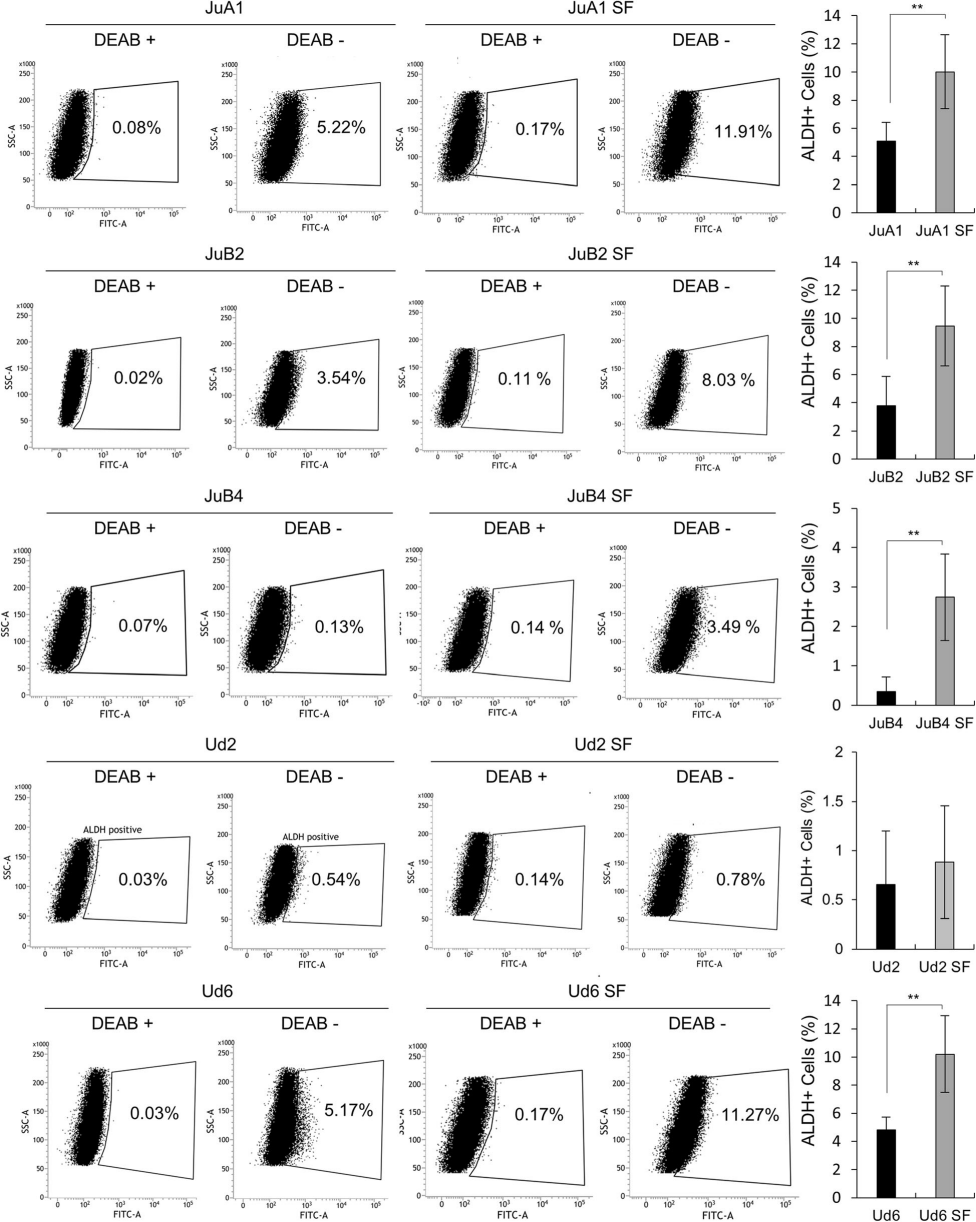
Flow cytometry analysis for ALDH activity. The percentages of ALDH positive cells are shown in each box. The bar graphs at the right end of each line indicate average percentages of ALDH^+^ cells with ±SD. The boxes were drown based on the result of negative control samples treated with DEAB

Since cancer stem cells have higher resistance to anti-cancer drugs, we analyzed sensitivities of HSA cell lines to doxorubicin and paclitaxel in normal and SF conditions [12, 13]. All cell lines cultured in SF condition had significantly higher resistance to both chemotherapeutic drugs although the extents vary between cell lines (Fig. 2). ALDH is known as one of the CSC markers highly associated with drug resistance capability, hence, ALDH activities were analyzed [51, 52]. Flow cytometry analysis revealed that the percentage of ALDH positive cells were significantly increased in SF condition except for Ud2 (Fig. 3).

These results suggest that HSA cells isolated by our SF culture method have CSC-like characteristics.

### Notch2 signal is required for HSA CSC-like cell survival in serum-free culture condition

**Fig. 4:**
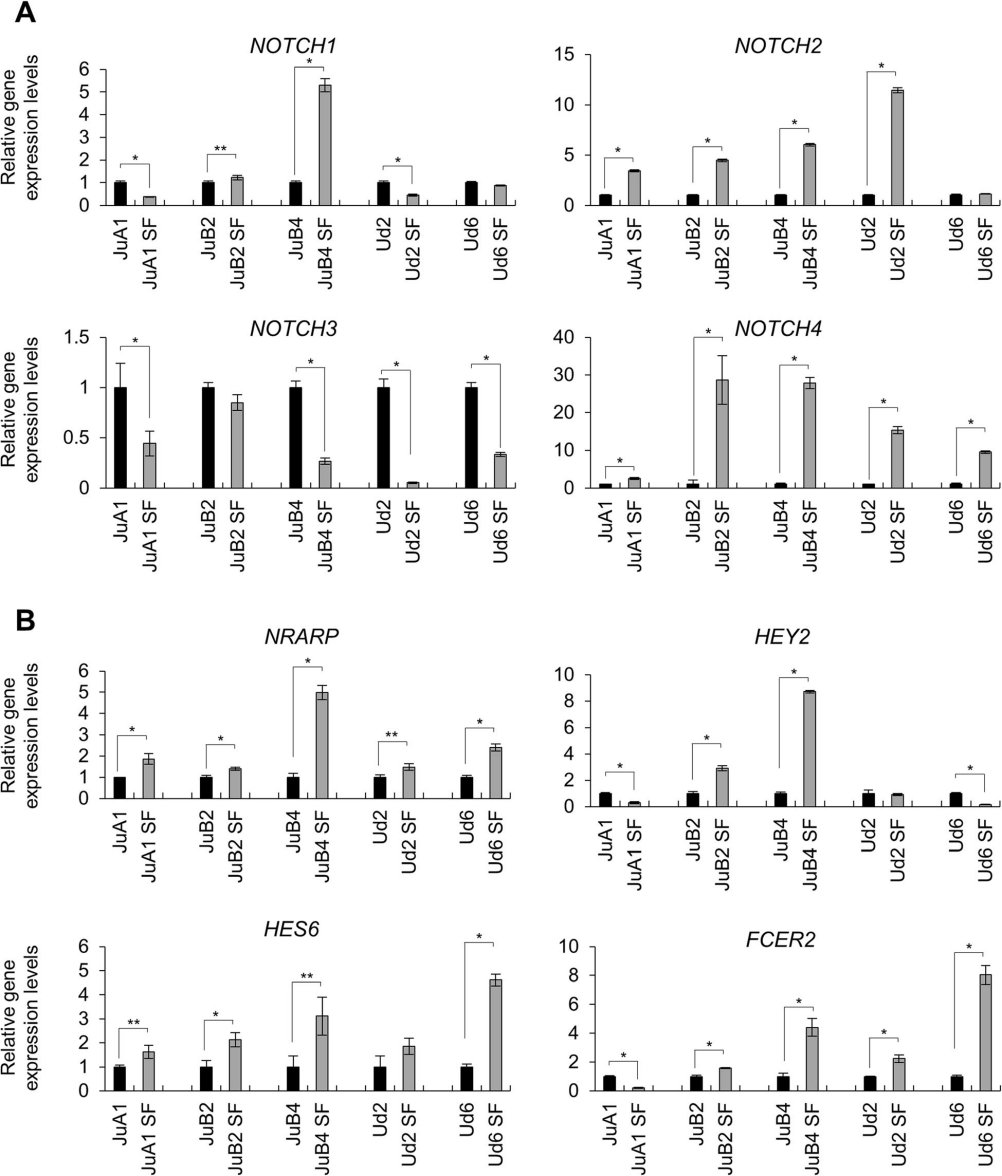
Gene expression levels of Notch receptors (**a**) and Notch target genes (**b**). *p < 0.01. **p < 0.05. Student’s *t* test. All samples were analyzed in triplicates and the scores are presented as means ± SD

**Fig. 5:**
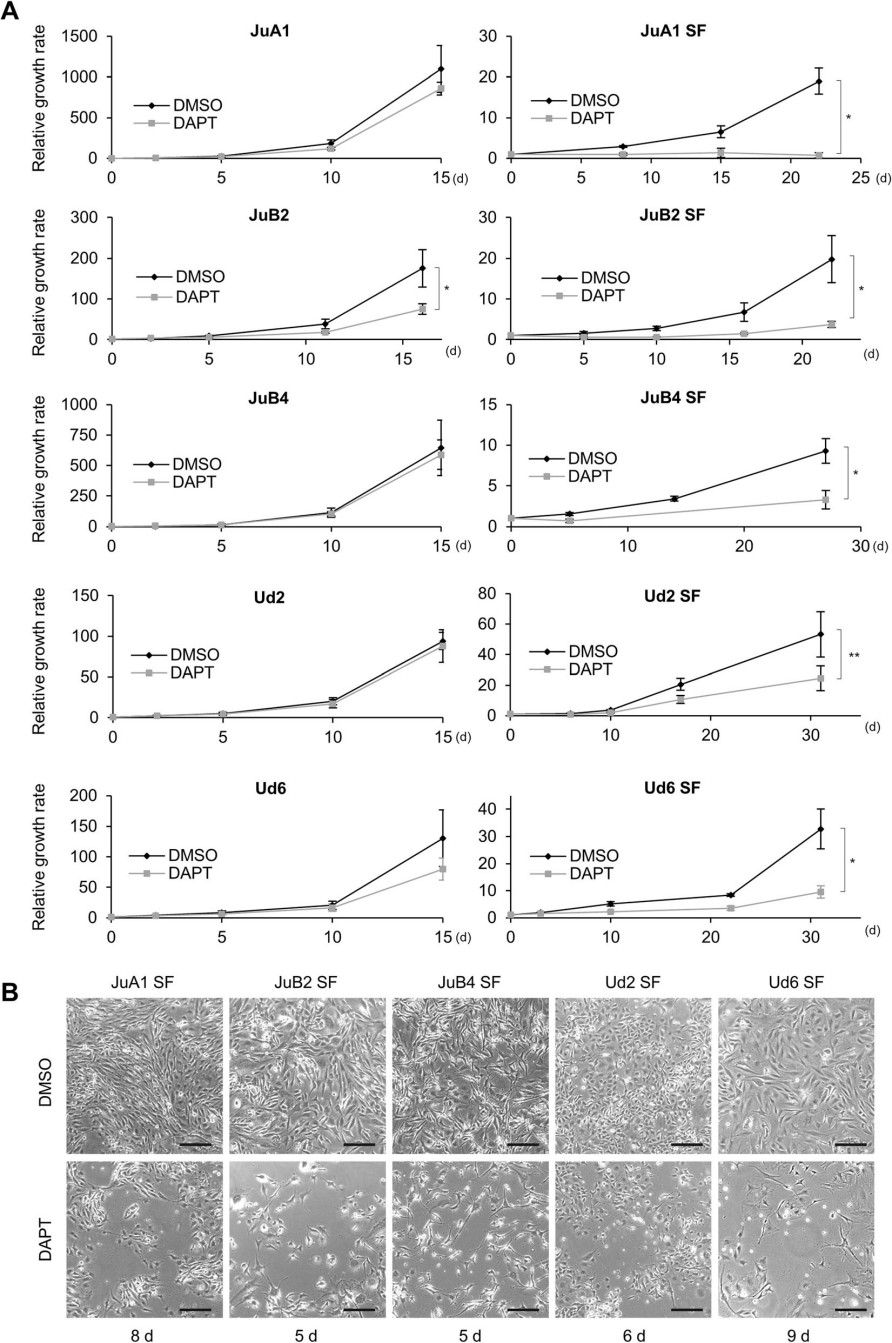
**a** Relative growth rate of HSA cell lines cultured in normal or SF condition treated with DMSO or DAPT. *p < 0.01. **p < 0.05. Student’s *t* test. All samples were analyzed in triplicates and growth curves are presented as means ± SD. **b** Representative images of HSA cell lines in serum-free condition treated with DAPT or not. Bars = 100 μm

We succeeded in isolating CSC-like cells from HSA cell lines but the genes nor the factors which are important for these cells’ survival were still unclear. Notch signal has been previously reported as a necessary signal transduction for tumor development, stem cell maintenance including CSCs, and angiogenesis [19–28]. Dysregulation of Notch signal has been associated with vascular tumors in humans and mice [30–32]. Thus, we tried to investigate its function in HSA cell lines. First, we analyzed gene expression levels of Notch receptors (*NOTCH1*, *NOTCH2*, *NOTCH3* and *NOTCH4*) and Notch target genes (*NRARP*, *HEY2*, *HES6* and *FCER2*) in normal and in SF conditions. *NOTCH2* and *NOTCH4* were upregulated in SF condition in all cell lines except for *NOTCH2* in Ud6, in addition, at least two target genes were expressed higher in SF condition than normal condition (Fig. 4a and b). To check the Notch2 function in HSA cell growth, we tried to inhibit the function with a γ-secretase inhibitor, DAPT. We tested DAPT for HSA cell lines at several concentration and found out that 20 μM DAPT was enough to repress Notch signal target gene expression (Additional file 4: Figure S4) and was therefore used for further experiments. In normal condition, all cell lines except for JuB2 did not show any significant decrease in growth rate after DAPT treatment when compared to the control (Fig. 5a). In contrast, all cell lines in SF condition had dramatically decreased growth rate after treatment with DAPT (Fig. 5a and b). These results suggest that Notch signal is required for HSA cell survival in SF condition.

**Fig. 6:**
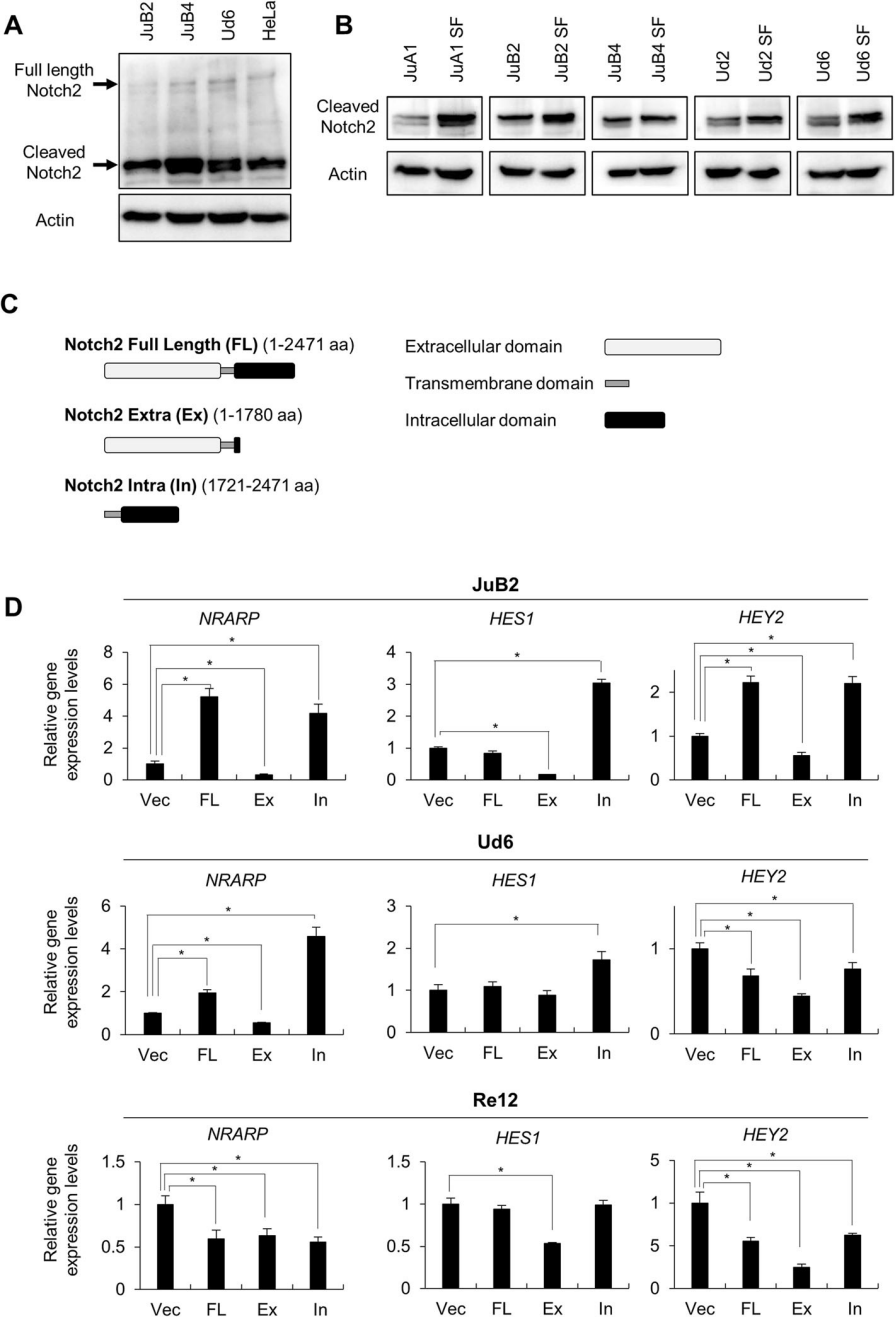
**a** Western blot analysis using anti-human Notch2 antibody. HeLa cell line was used as a positive control. **b** Notch2 protein expression levels in HSA cell lines cultured under the normal or serum-free condition, **c** Conceptual diagram of Notch2 protein and its mutants. **d** Gene expression levels of Notch target genes. Vec = cells transfected the empty vector. FL = cells overexpressing full length of Notch2. Ex = cells overexpressing dominant negative form of Notch2. In = cells overexpressing constitutive active form of Notch2. Gene expression levels of Vec were set to 1. *p < 0.01. **p < 0.05. Dunnett’s test. Samples for gene expression analysis were analyzed in triplicates and the scores are presented as means ± SD

**Fig. 7:**
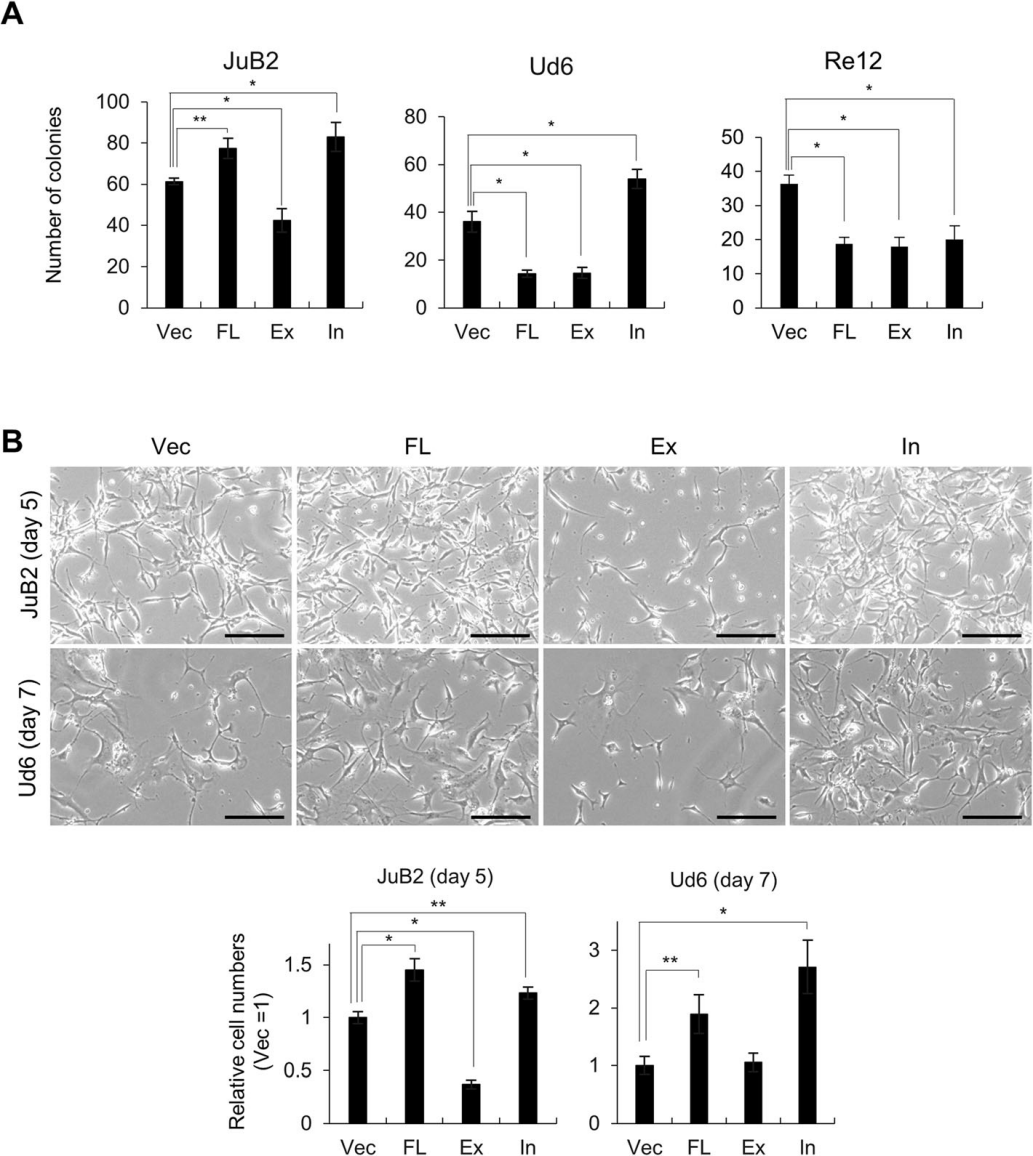
**a** Colony numbers of HSA cell lines overexpressing Notch2 vector constructs. **b** (Top) Representative images of JuB2 and Ud6, 5 and 7 days after starting serum-free culture, respectively. Bars = 100 μm. (Bottom) Relative cell numbers of JuB2 and Ud6. Number of Vec was set to 1. *p < 0.01. **p < 0.05. Dunnett’s test. The scores are presented as means ± SD

Although *NOTCH2* and *NOTCH4* were upregulated in SF condition (Fig. 4a), 2^-^^ΔCt^ values of *NOTCH2* was much higher than that of *NOTCH4* (Table 3). In addition, Notch2 specific target *FCER2* was upregulated, which encouraged us to analyze the effects of Notch2 in HSA cell lines. Prior to examining Notch2 protein expression, we confirmed that anti-human Notch2 antibody can cross-react with canine Notch2 (Fig. 6a). Notch2 protein expression was higher in SF condition than the normal condition (Fig. 6b). Based on previous research, we constructed lentiviral vectors to stably express Notch2 full length form (FL), dominant negative form (Ex) and constitutive active form (In) in JuB2, Ud6, and Re12 in normal culture condition (Fig. 6c) [46]. Notch2 expression from these constructs were confirmed using anti-FLAG antibody and anti-Notch2 antibody (Additional file 5: Figure S5). After establishment of stable cell lines, we analyzed Notch signal target genes, *NRARP*, *HES1* and *HEY2* to confirm the functions of wild type and mutant Notch2 in stable cell lines. JuB2 and Ud6, where we succeeded in isolating CSC-like cells with SF culture, showed significantly higher expressions of these target genes in cells overexpressing FL and In, and significantly lower expressions in cells overexpressing Ex compared to vector controls (Vec) (Fig. 6d). In contrast, Re12 did not show any upregulation of the target genes’ expression in cells overexpressing FL and In although Ex overexpression in Re12 repressed them. Next, we checked the effects of Notch2 on clonogenicity using CFA, which resulted in the significant increase of colony numbers in In-overexpressing cells and significant decrease in Ex-overexpressing cells in both JuB2 and Ud6 cells, on the other hand, the colony formation was significantly decreased in Re12 overexpressing any types of Notch2 (Fig. 7a). Lastly, we checked whether Notch2 inhibition or activation affected CSC-like cell numbers after changing culture condition from normal to SF. As a result, Notch2 inhibition by Ex overexpression significantly decreased the number of viable cells in JuB2, while, Notch2 activation by FL- and In-overexpression resulted to significantly increased number of viable cells in JuB2 and Ud6 cell lines (Fig. 7b). These results suggest that Notch2 signal is required for the maintenance of HSA CSC-like cells.

**Table 3:** 2^-ΔCt^ values of *NOTCH2* and *NOTCH4*

2^-ΔCt^	JuA1	JuA1 SF	JuB2	JuB2 SF	JuB4	JuB4 SF	Ud2	Ud2 SF	Ud6	Ud6 SF
*NOTCH2*	0.0179	0.0618	0.2430	1.0862	0.2346	1.4135	0.0828	0.9478	0.9553	1.0772
*NOTCH4*	0.0606	0.1553	0.0002	0.0053	0.0009	0.0240	0.0052	0.0801	0.0009	0.0084

### Notch2 is highly expressed in clinical HSA cases

**Fig. 8:**
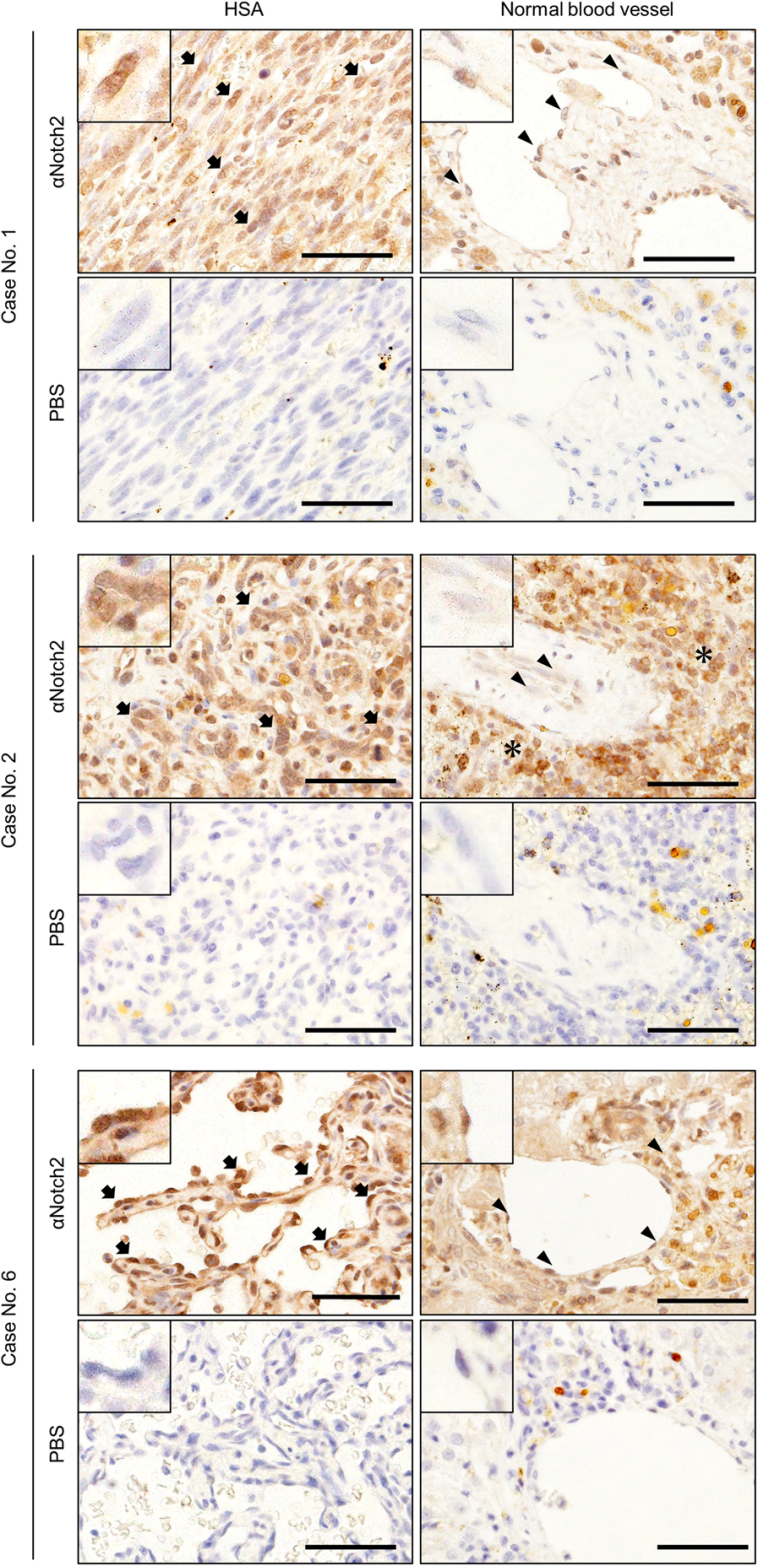
Immunohistochemistry analysis for clinical HSA cases using anti-human Notch2 antibody. Insertion indicated the magnified views of tumor cells or normal endothelial cells. Arrows = neoplastic cells. Arrow heads = endothelial cells in normal blood vessels in the same slides. Asterisks = lymphocytes. Bars = 50 μm

We analyzed Notch2 expression in clinical HSA cases with IHC. Twelve HSA cases were tested and all cases were positive for anti-Notch2 antibody (Fig. 8). Stronger staining intensities were observed in neoplastic cells compared to the endothelial cells of normal blood vessels in the same slide. The staining intensities of tumor cells were at the same level with lymphocytes, a positive control for Notch2 (Fig. 8 case No.2). These results suggest that Notch2 is also active in clinical HSA cases.

## Discussion

In this study, we found that Notch2 is a key factor for the maintenance of HSA CSC-like cells. Isolated cells from our serum-free culture method had CSC-like characteristics such as upregulation of undifferentiated endothelial cell markers, high clonogenicity, high drug resistance, and increased ALDH^+^ cell population. Constitutive activation of Notch2 resulted to an increase of the number of cells with high clonogenicity in normal culture condition and cells which can survive in SF condition, while Notch2 inhibition caused opposite effects. Furthermore, Notch2 was found to be highly expressed and active in clinical HSA cases.

We succeeded in culturing CSC-like cells from Ju and Ud cell lines but, unfortunately, we were not able to isolate CSC-like cells from Re cell lines (Re12 and Re21). Furthermore, any types of Notch2 overexpression in Re12 repressed downstream gene expressions and colony formation. Re cell lines were derived from HSA in the right atrium of the heart of a Golden retriever [33, 53]. Several reports have indicated that HSA of Golden retrievers has different characteristics from those of other breeds [54–56]. Tamburini et al. have demonstrated that significant upregulation of *VEGFR1* was observed in HSA from Golden retrievers compared to other breeds [54]. Vegfr1 is a tyrosine kinase receptor which can bind to an angiogenic factor Vegfa. It was thought as a decoy receptor for Vegfr2 which has higher tyrosine kinase activity than Vegfr1 and works as a major transducer of Vegfa. However, it has recently been reported that Vegfr1 can also transduce signals and stimulates tumor growth and metastasis [57]. Vegfr2 has different signal transductions from Vegfr1 and induces angiogenesis specifically by regulating Ets1 transcription factor [58]. Those different signal transductions and transcriptional regulation probably give different characteristics to HSA in Golden Retrievers. Further research comparing the molecular biology of HSA from Golden Retrievers and other breeds is required.

Notch2 is one of the Notch signal receptors and has been reported as an important factor both for tumorigenesis and stem cell maintenance. Active mutations in *NOTCH2* are involved in developments of diffuse large B cell lymphoma and marginal zone B-cell lymphoma [59–62]. In human hepatocellular carcinoma, Notch2 regulates the stemness of liver CSCs via upregulation of *NRARP*, *HES1* and *HES6* [63]. Notch2 is also highly upregulated in pancreatic cancer-stem cells [64]. In our study, activation of Notch2 by FL and In in JuB2 increased the number of colonies observed in CFA and survival cell numbers in SF condition, and Notch signal inhibition by Ex indicated opposite effects. Ud6 overexpressing In had similar results with JuB2, but Ud6 cells overexpressing FL or Ex showed different results wherein the cells overexpressing FL had decreased number of colonies while those overexpressing Ex showed no difference with vector control even after changing to SF culture medium. The discrepancy between JuB2 and Ud6 both of which expressed FL is probably resulted from the difference in the amount of ligands which can trigger Notch signals. In, the constitutive active form, overexpression showed increase in number of colonies and survival of cells in either experiment. There may not be enough number of cells secreting ligands in Ud6 in normal cell culture condition. Negative feedback system of Notch signaling may also be related [65]. In Ud6 cells overexpressing Ex, other factors such as other Notch receptors may compensate for the loss of function of Notch2 in SF condition. Notch2 may be important for stemness maintenance in HSA, however, we could not identify its responsible ligands and direct targets. Further experiments which focus on the targets of Notch2 to maintain stemness in HSA are warranted. Also, since *NOTCH4* was also highly upregulated in SF condition, it is worth analyzing its function for cell survival in SF condition.

Although we can’t exclude the possibility that Notch signal was just artificially activated by stimulating the Fgf pathway and gelatin coating, we speculate that our culture condition imitates the microenvironment for hemangiosarcoma cancer stem cells. In general, microenvironment is required to maintain stem cells in their undifferentiated state by transducing signals and/or by providing cytokines. Neoplastic cells can be differentiated when they go out of the microenvironment [12]. Based on this nature, imitating suitable microenvironment is necessary to culture cancer stem cell-like cells even in vitro condition. However, further experiments to analyze Fgf pathway activity including Fgf receptor and ligand expression in tumor cells and in microenvironment components such as inflammatory cells and fibroblasts are required.

## Conclusions

In conclusion, we succeeded in isolating CSC-like cells in HSA using our own method, and demonstrated that Notch2 is a key factor for the maintenance of HSA CSC-like cells. Our study can encourage further stem cell research in HSA and may provide a way to develop effective treatments targeting CSCs.

## Additional files

Additional file 1: **Figure S1.** Standard curves for each primer. Slope was used to calculate primer efficiencies. (TIF 801 kb)

Additional file 2: **Figure S2.** Standard curves for each primer. Slope was used to calculate primer efficiencies. (TIF 794 kb)

Additional file 3: **Figure S3.** Results of geNorm analysis for reference gene candidates. To determine optimal number of reference genes, 0.15 *V* value was used as the cut-off value as Vandesompele et al. [38] recommended. (TIF 1355 kb)

Additional file 4: **Figure S4.** Gene expression levels of Notch signal target genes. HSA cells treated with DMSO were set to 1. *p < 0.01. **p < 0.05. Dunnett’s test. (TIF 576 kb)

Additional file 5 **Figure S5.** Western blot analysis to detect Notch2 constructs expressions using anti-FLAG antibody (A) and anti-Notch2 antibody (B). Since anti-Notch2 antibody that we used can detect the Notch2 intracellular domain, the Notch2 Ex was not detected. Vec = cells transfected the empty vector. FL = cells overexpressing full length of Notch2. Ex = cells overexpressing dominant negative form of Notch2. In = cells overexpressing constitutive active form of Notch2. (TIF 1028 kb)

## Abbreviations

ALDH: Aldehyde dehydrogenase
bFGF: Basic fibroblast growth factor
CFA: Colony formation assay
CnAOEC: Canine Aortic Endothelial Cells
CSCs: Cancer stem cells
DAPT: N-[N-(3,5-Difluorophenacetyl)-L-alanyl]-S-phenylglycine t-butyl estel
DEAB: N,N-diethylaminobenzaldehyde
D-MEM: Dulbecco’s Modified Eagle’s Medium
DMSO: Dimethyl sulfoxide
EGF: Epidermal growth factor
Ex: Notch2 dominant negative form
FL: Notch2 full length form
HSA: Hemangiosarcoma
IHC: Immunohistochemistry
In: Notch2 constitutive active form
PBS: Phosphate buffered saline
PCR: Polymerase chain reaction
PVDF: Polyvinylidene difluoride membrane
RiPA buffer: Radioimmunoprecipitation buffer
RT: Room temperature
RT-qPCR: Reverse transcription quantitative polymerase chain reaction
SDS: Sodium dodecyl sulfate
SF: Serum-free
SIN: Self-inactivating
TBST: Tris-buffered saline containing 0.05% Tween 20
UPDW: UltraPure DNase/RNase-free distilled water
