# Supplementary figures and images for: Notch2 signal is required for the maintenance of canine hemangiosarcoma cancer stem cell-like cells
Source: BMC Vet Res. 2018 Oct 3;14:301. doi: 10.1186/s12917-018-1624-8 (PMC6171240; doi:10.1186/s12917-018-1624-8)

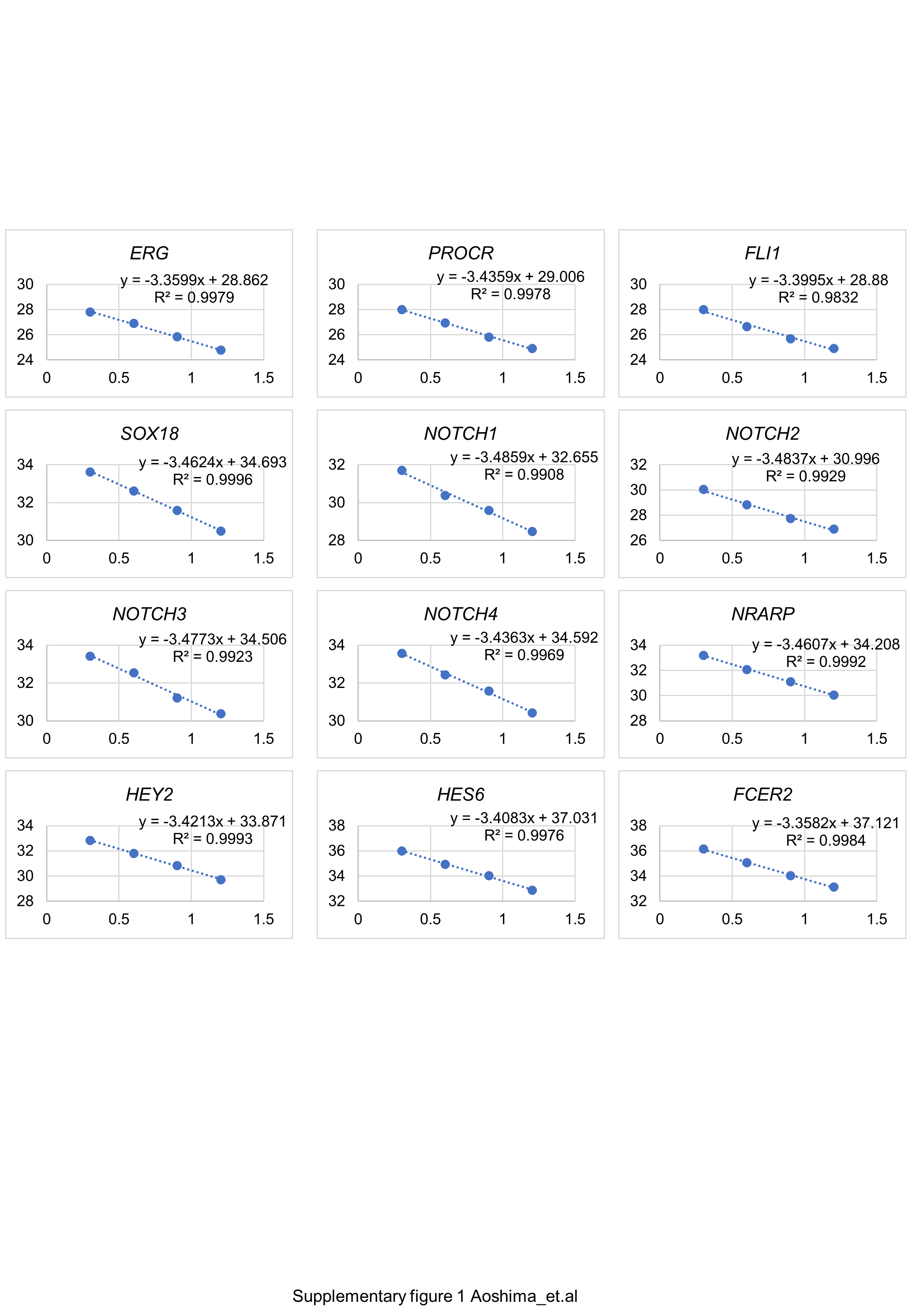

Supplement: Supplementary file 1 — Figure S1. Standard curves for each primer. Slope was used to calculate primer efficiencies. (TIF 801 kb) [file 12917_2018_1624_MOESM1_ESM.tif]

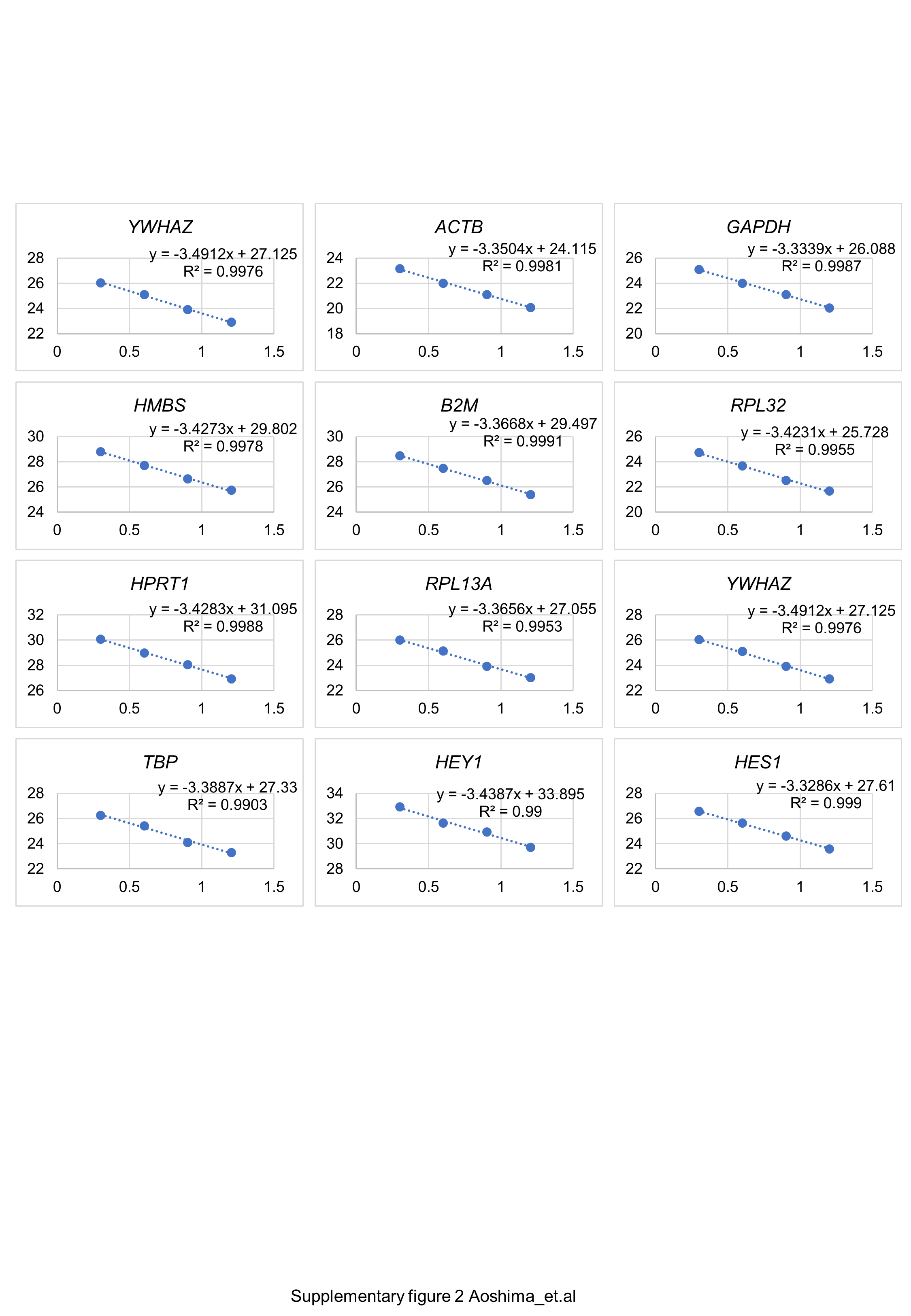

Supplement: Supplementary file 2 — Figure S2. Standard curves for each primer. Slope was used to calculate primer efficiencies. (TIF 794 kb) [file 12917_2018_1624_MOESM2_ESM.tif]

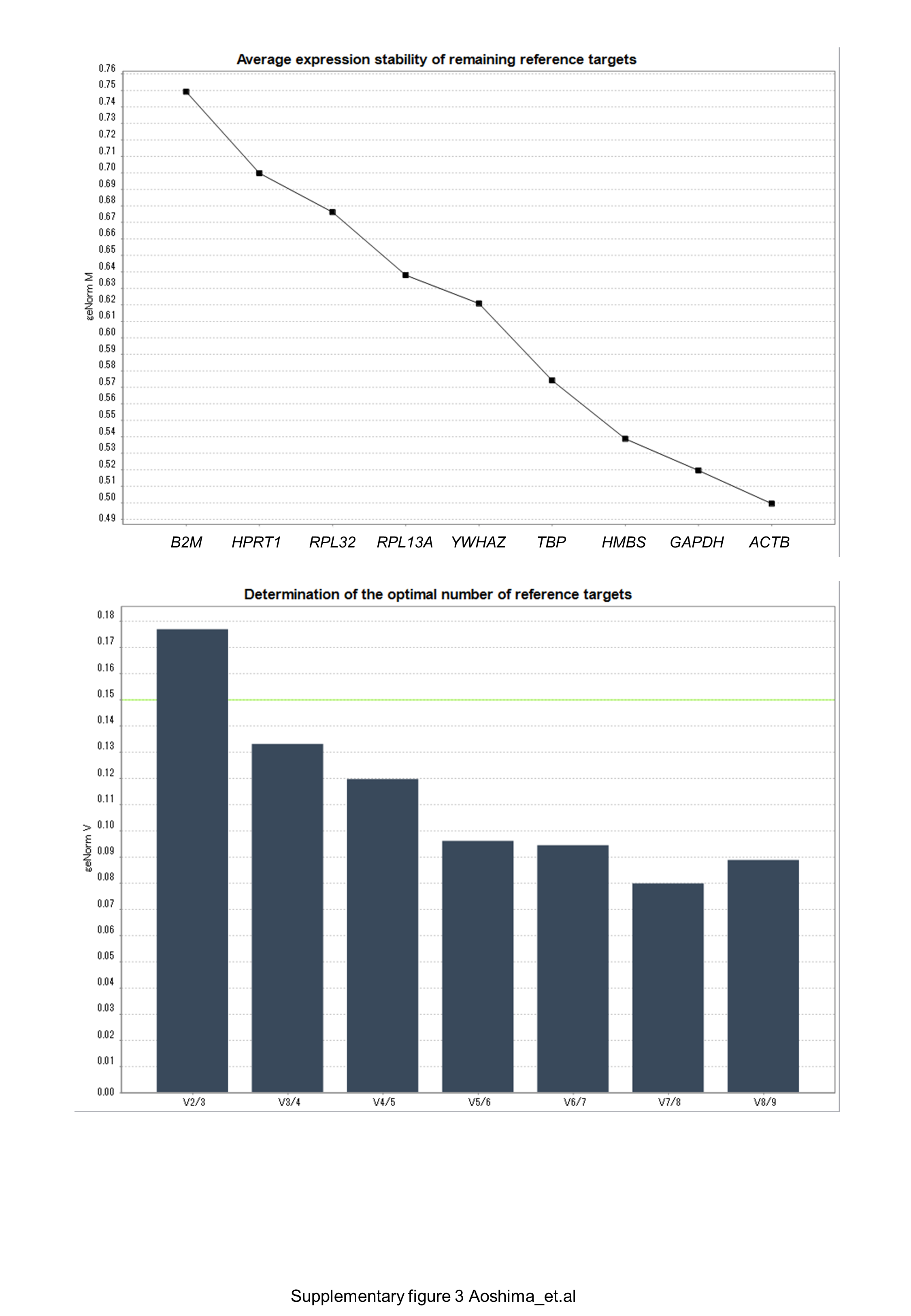

Supplement: Supplementary file 3 — Figure S3. Results of geNorm analysis for reference gene candidates. To determine optimal number of reference genes, 0.15 V value was used as the cut-off value as Vandesompele et al. [38] recommended. (TIF 1355 kb) [file 12917_2018_1624_MOESM3_ESM.tif]

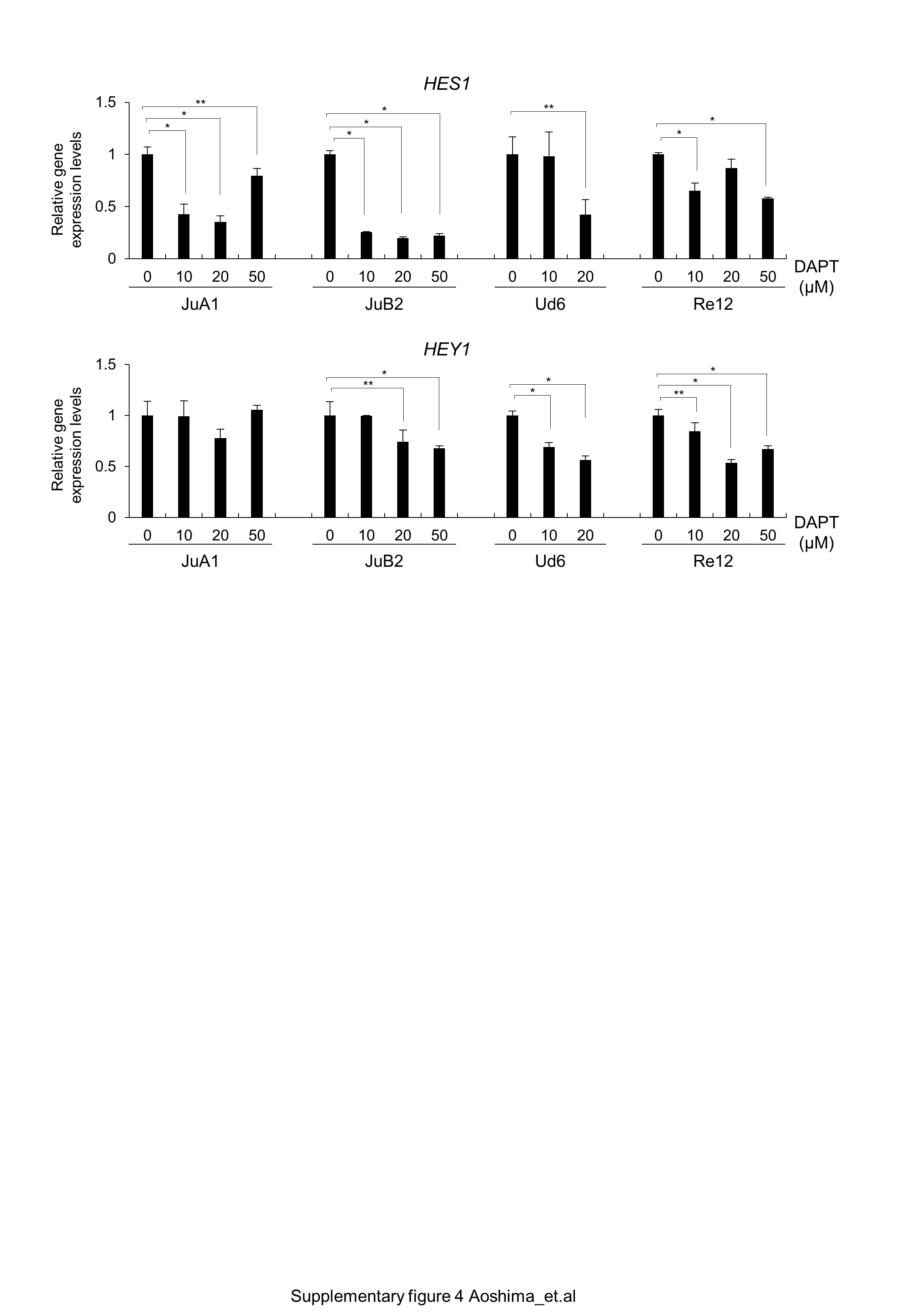

Supplement: Supplementary file 4 — Figure S4. Gene expression levels of Notch signal target genes. HSA cells treated with DMSO were set to 1. *p < 0.01. **p < 0.05. Dunnett’s test. (TIF 576 kb) [file 12917_2018_1624_MOESM4_ESM.tif]

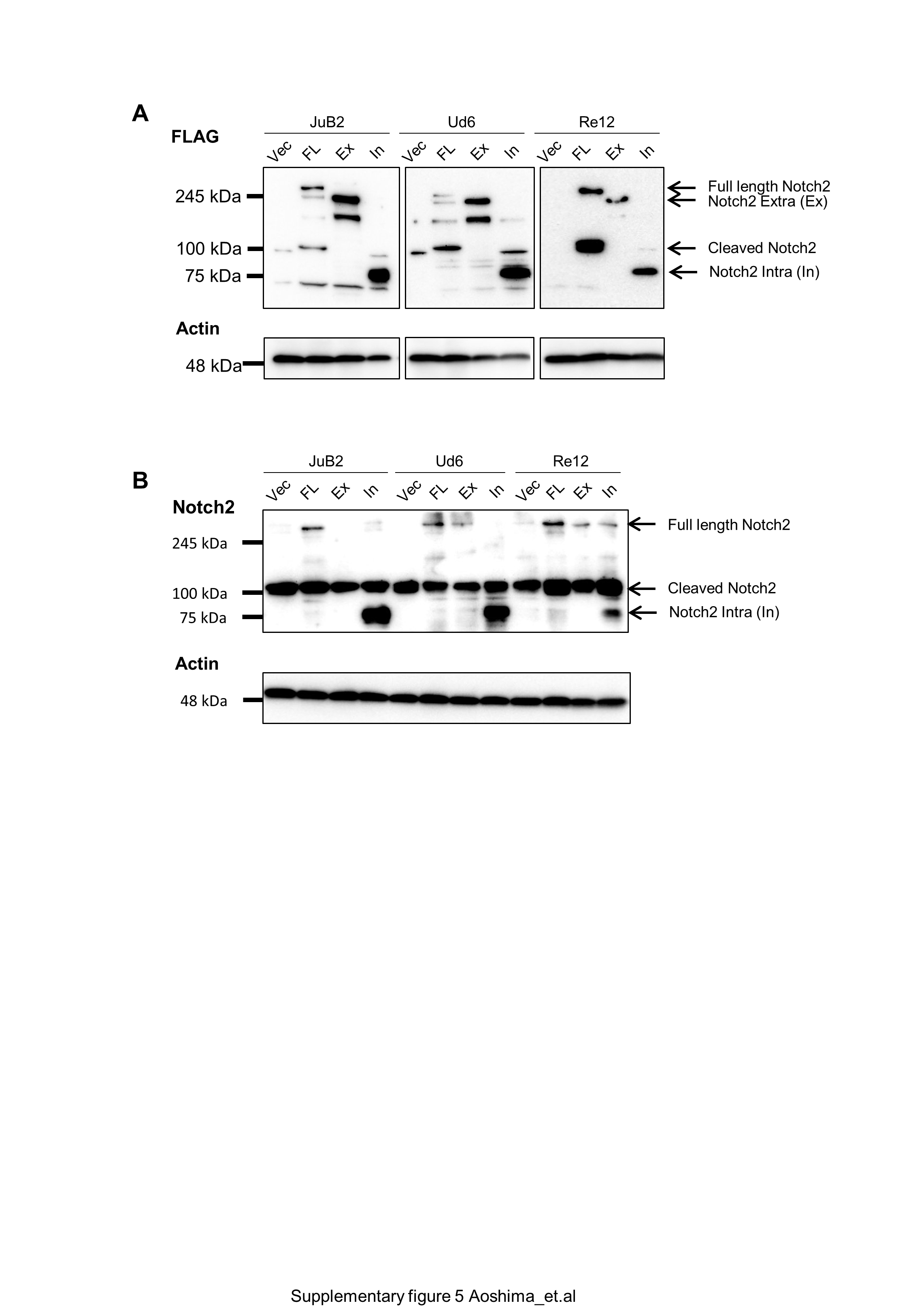

Supplement: Supplementary file 5 — Figure S5. Western blot analysis to detect Notch2 constructs expressions using anti-FLAG antibody (A) and anti-Notch2 antibody (B). Since anti-Notch2 antibody that we used can detect the Notch2 intracellular domain, the Notch2 Ex was not detected. Vec = cells transfected the empty vector. FL = cells overexpressing full length of Notch2. Ex = cells overexpressing dominant negative form of Notch2. In = cells overexpressing constitutive active form of Notch2. (TIF 1028 kb) [file 12917_2018_1624_MOESM5_ESM.tif]
